# T-cell lymphoblastic lymphoma of the breast: A retrospective observational study and literature review

**DOI:** 10.1097/MD.0000000000047991

**Published:** 2026-03-13

**Authors:** Wei Bian, Jun Yuan, Yufeng Fei, Jinjuan Feng, Xu Zhang, Sili Jin, Yi Yang

**Affiliations:** a Department of Radiology, Jiaxing Maternity and Child Health Care Hospital, College of Medicine, Jiaxing University, Jiaxing Women and Children’s Hospital Affiliated to Wenzhou Medical University, Jiaxing, China; b Department of Pathology, Jiaxing Maternity and Child Health Care Hospital, College of Medicine, Jiaxing University, Jiaxing Women and Children’s Hospital Affiliated to Wenzhou Medical University, Jiaxing, China; c Department of Radiology, Ningbo City Yinzhou District Third Hospital, China; d Breast Tumor Center, Ruijin Hospital Affiliated to Shanghai Jiao Tong University School of Medicine, Shanghai, China; e Department of Breast, Jiaxing Maternity and Child Health Care Hospital, College of Medicine, Jiaxing University, Jiaxing Women and Children’s Hospital Affiliated to Wenzhou Medical University, Jiaxing, China.

**Keywords:** breast, lymphoblastic, lymphoma, non-Hodgkin, T-cell

## Abstract

This study aims to explore the diagnosis and treatment of T-cell lymphoblastic lymphoma (T-LBL) of the breast. We retrospectively analyzed the clinical data of a patient with breast T-LBL jointly managed by our hospital and Ruijin Hospital, Shanghai. A systematic literature review was conducted across PubMed, Web of Science, Embase, Cochrane Library, China Biomedical Literature Database, China national knowledge infrastructure, Wanfang, and Wipro databases up to May 10, 2024, using Boolean operators (e.g., (“T-cell lymphoblastic lymphoma” OR “T-LBL”) AND (“breast” OR “mammary”)). Including our case, 11 patients were studied. All were female (mean age 31.81 ± 11.97 years). Five cases involved both breasts, 6 were unilateral. Ten cases had extramammary involvement. Immunohistochemistry was positive for both terminal deoxynucleotidyl transferase and cluster of differentiation (CD3) in 9 breast lesions. Imaging included ultrasound (n = 6), mammography (n = 6), magnetic resonance imaging (n = 2), and positron emission tomography–computed tomography (n = 2). Most patients (n = 10) received chemotherapy, with some supplemented by radiotherapy and intrathecal prophylaxis. Breast T-LBL is exceedingly rare. Accurate diagnosis is crucial for management. Future prospective studies are needed to optimize diagnostic and therapeutic strategies.

## 1. Introduction

Lymphoblastic lymphoma, a rare and aggressive subtype of non-Hodgkin’s lymphoma, originates from precursor lymphoblastoid cells at various stages of differentiation. It is categorized into B-cell and T-cell lineages, with T-cell lymphoblastic lymphoma (T-LBL) constituting ~70% to 80% of cases. T-LBL exhibits a distinct epidemiological profile, predominantly affecting adolescents and young adults, with a notable male predominance. It commonly presents with a rapidly enlarging mediastinal mass and frequent involvement of lymph nodes, while primary extranodal manifestations, such as in the breast, are exceedingly rare.

Immunophenotypically, T-LBL blasts typically express immature T-cell markers. The most consistent and characteristic markers include cytoplasmic cluster of differentiation (CD3; cCD3), CD7, and terminal deoxynucleotidyl transferase (TdT).^[1]^ CD1a, CD2, CD5, and CD4/CD8 (either as double-positive or double-negative) are variably expressed, reflecting the stage of thymic differentiation.^[2]^ It is also common to observe co-expression of the myeloid-associated markers CD13 or CD33. This immunophenotypic profile is crucial for distinguishing T-LBL from other mature T-cell lymphomas and for confirming its precursor cell origin.

The similarities in morphology, immunophenotype, and clinical behavior between T-LBL and acute T-cell lymphoblastic leukemia (T-ALL) have led the 2022 World Health Organization (WHO) classification to regard them as a single biological entity manifesting across a disease spectrum. The operational distinction remains based on the extent of bone marrow involvement: a blast proportion of <25% is classified as T-LBL, while ≥25% defines T-ALL. Primary involvement of the breast by lymphoblastic lymphoma represents an exceptional clinical scenario.^[3]^ This article analyzes the clinical data of patients with T-LBL treated at our hospital and Shanghai Ruijin Hospital, aiming to enhance our understanding of T-LBL in breast tissues.

## 2. Materials and methods

### 2.1. Clinical information

**Figure 1.**
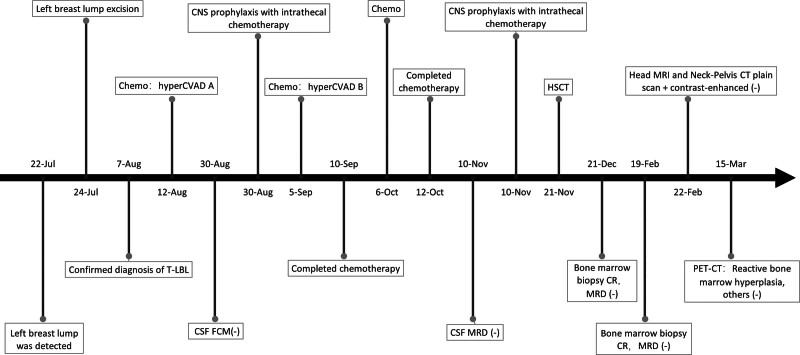
The diagnosis and treatment process of our case. hyperCVAD A: cyclophosphamide 500 mg every 12 hours on days 1 to 3 + doxorubicin 4 mg on day 4 and day 11 + liposomal amphotericin B 50 mg on day 4+ dexamethasone 40 mg on days 1 to 4 and 11 to 14; intrathecal chemotherapy: dexamethasone 5 mg + cytarabine 50 mg + methotrexate 10 mg; hyperCVAD B: methotrexate 1680 mg on day 1 + cytarabine 2500 mg every 12 hours on days 2 to 3. Chemo = chemotherapy, CNS = central nervous system, CSF = cerebrospinal fluid, FCM = flow cytometry, HSCT = hematopoietic stem cell transplantation, T-LBL = T-cell lymphoblastic lymphoma.

From July 2023 to April 2024, Jiaxing Maternity and Child Health Care Hospital and Shanghai Ruijin Hospital collaborated to diagnose and treat a singular case of breast T-LBL. The patient, a 45-year-old female, incidentally discovered a walnut-sized lump in her left breast ~2 weeks prior, presenting with mild tenderness, absence of nipple discharge, localized erythema and edema, and no systemic symptoms. Upon physical examination, bilateral breast symmetry was noted, with a 4 cm × 3 cm firm mass situated laterally in the left breast exhibiting indistinct borders, limited mobility, and absence of tenderness on palpation. The right breast revealed no palpable abnormalities, with convex nipples, absence of discharge, and unremarkable axillary, clavicular, and cervical lymph nodes. Subsequent excision of the left breast mass revealed histological features suggestive of lymphomatous involvement, with examination of 3 left axillary sentinel lymph nodes demonstrating no neoplastic infiltration. Subsequent referral to Ruijin Hospital, affiliated with Shanghai Jiaotong University School of Medicine, facilitated comprehensive diagnostic evaluations including pathology, molecular profiling, bone marrow aspiration, and ^18^F-Fluorodeoxyglucose positron emission tomography-computed tomography (^18^F-FDG PET-CT) imaging, consolidating the diagnosis of breast T-LBL. Treatment encompassed 3 cycles of chemotherapy with adjunctive nutritional and anti-infective support, lumbar puncture with intrathecal therapy, and allogeneic stem cell transplantation. Follow-up assessments, comprising bone marrow aspiration and ^18^F-FDG PET-CT scans, documented complete remission. A schematic representation depicting the patient’s diagnostic and therapeutic journey was devised to illustrate the course of clinical management (Fig. 1).

### 2.2. Literature search

To ensure transparency and reproducibility, a systematic literature search was conducted across multiple databases, including PubMed, Web of Science, Embase, Cochrane Library, China Biomedical Literature Database, China National Knowledge Infrastructure, Wanfang, and Wipro, up to May 10, 2024. The search strategy employed Boolean operators, combining key terms such as (“T-cell lymphoblastic lymphoma” OR “T-LBL”) AND (“breast” OR “mammary”) in English databases and their corresponding Chinese translations in Chinese databases. Advanced filters were applied to restrict results by publication type, date range, and human studies. The selection criteria included case reports, clinical studies, and reviews relevant to breast T-cell lymphoblastic lymphoma, while unrelated lymphoma types, non-breast involvement, and duplicate publications were excluded. This methodology was designed in accordance with established systematic review practices to ensure a comprehensive and replicable search process.

The inclusion criteria for identified cases encompassed patients diagnosed with histopathologically confirmed T-LBL, accompanied by comprehensive clinical, imaging, and pathological documentation. The exclusion criteria involved the exclusion of T-ALL cases and literature lacking myelocentesis procedures. Furthermore, duplicate cases originating from identical authors, institutions, research entities, and databases were omitted from consideration.

## 3. Results

### 3.1. Imaging results

**Figure 2.**
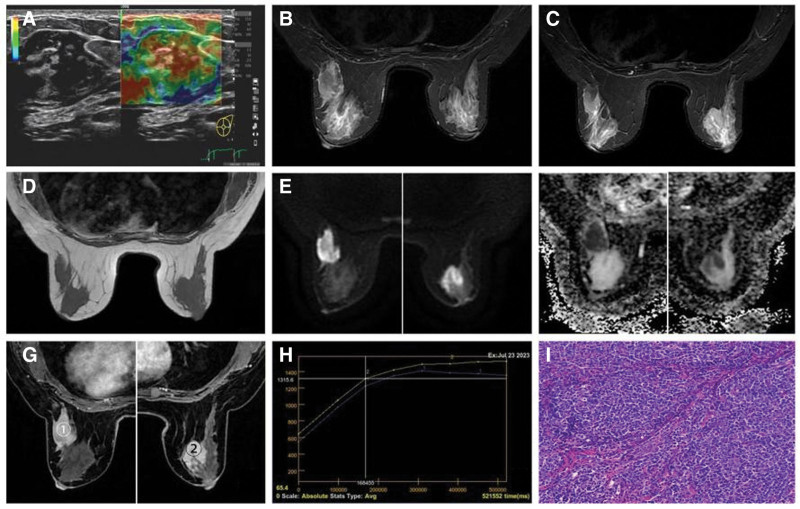
Preoperative ultrasound, MRI and postoperative pathological results. (A) Breast ultrasound: the left breast exhibited heterogeneous hypoechoic lesions with indistinct borders and irregular morphology. Elasticity imaging revealed a grade 4 lesion. (B–H) E–G were composite images of the central plane of both breasts in the prone position) prone position breast MRI images demonstrated irregular lesions in the outer quadrant of the left breast and inner quadrant of the right breast with unclear borders. On T2-weighted imaging, the lesion in the left breast displayed isointensity in the center and slightly hyperintensity at the edges (B, C), while the lesion in the right breast showed slightly higher signal intensity (B, C). T1-weighted imaging showed iso-intensity (D), diffusion-weighted imaging showed hyperintensity (E), and apparent diffusion coefficient (ADC) mapping revealed significantly hypointensity with an ADC value of ~0.58 × 10^−3^ mm^2^/s (F). Following contrast enhancement, both breast lesions exhibited heterogeneous enhancement (G), and the time-intensity curve (TIC) demonstrated a plateau pattern (H). (I) Pathological examination of the lesion in the left breast after surgery revealed tumor cells with small volume, oval-shaped nuclei, indistinct nucleoli, and frequent nuclear division under high magnification. ADC = apparent diffusion coefficient, MRI = magnetic resonance imaging, TIC = time-intensity curve.

Ultrasonography assessment of both breasts revealed the presence of scattered hyperechoic strips interconnected within the internal tissues, locally fused to create a nodular configuration. Additionally, a 4.1 cm × 3.8 cm × 2.0 cm heterogeneous hypoechoic lesion was observed in the left lateral breast, displaying poorly defined margins, irregular morphology, and grade I blood flow, characterized by an resistivity index (RI) of 0.60. Furthermore, elastography demonstrated a grade 4 for this lesion (see Fig. 2A). In the left axilla, elliptical hyperechoic structures, measuring 1.1 cm × 0.8 cm and 0.9 cm × 0.7 cm with abundant medulla, were identified. No significantly enlarged lymph nodes were noted in the right axilla, and there were no evident abnormal echoes detected in the soft tissues of either axilla. Overall findings indicate bilateral breast mastopathy, a left breast mass with an elastography grade of 4, and a breast imaging reporting and data system category 0 on ultrasound assessment. Furthermore, lymph nodes were observed in the left axilla.

Mammographic imaging of both breasts revealed no notable masses.

A breast magnetic resonance imaging (MRI) unveiled an irregularly shaped mass measuring ~4.4 cm × 3.9 cm in the lateral quadrant of the left breast, exhibiting unclear borders and presenting with isohigh signal intensity on T2-weighted images (Fig. 2B, C), iso-signal on T1-weighted images (Fig. 2D), high signal on diffusion-weighted imaging (DWI; Fig. 2E), and low apparent diffusion coefficient (ADC) value of 0.58 × 10^−3^ mm^2^/s (see Fig. 2F). This mass demonstrated heterogeneous enhancement (Fig. 2G) and a plateau in the time-intensity curve (TIC) post-enhancement (Fig. 2H). Similarly, an irregularly shaped mass measuring around 2.0 cm × 2.3 cm with indistinct borders was detected in the inner lower quadrant of the right breast, displaying an ADC value of 0.58 × 10^−3^ mm^2^/s (Fig. 2F), uneven enhancement (Fig. 2G), and a plateau in the TIC (Fig. 2H). Furthermore, an irregularly shaped mass of similar characteristics measuring about 2.0 cm × 2.3 cm was observed in the lower quadrant of the right breast, with MRI signal patterns across various sequences, ADC values, enhancement patterns, and TIC curves consistent with those of the mass in the left breast. No distinct abnormal masses or mass-like enhancements were observed in the remaining bilateral breast tissues. MRI examination findings: bilateral breast masses designated as breast imaging reporting and data system 4B. Absence of bilateral nipple or skin infiltration noted. Visualization of lymph nodes in the left axilla within the imaging field, demonstrating no signs of enlargement.

Pre-chemotherapy ^18^F-FDG PET-CT findings: Postoperative changes in the left breast following resection, with no detectable FDG-avid lesions. Slightly elevated metabolic activity noted in the medial quadrant of the right breast, potentially attributable to physiological uptake. Several small lymph nodes in the left axilla, each measuring ~0.8 cm, exhibited increased metabolism with a maximum standardized uptake value (SUVmax) of ~5.1. Initially considered as inflammatory in nature, lymphoma infiltration was ruled out through close monitoring. Subsequent to chemotherapy, post-treatment ^18^F-FDG PET-CT findings revealed diffuse and uniform elevation of metabolic activity in the central bone marrow, with an SUVmax of 2.9. This was deemed likely due to reactive hyperplasia of the bone marrow. No conspicuous hypermetabolic lesions were observed in the overall body imaging following the treatment.

### 3.2. Pathology and bone marrow puncture results

Upon completion of all required examinations and exclusion of contraindications to surgery, a clinical diagnosis of stage IIA malignant tumor localized in the outer lower quadrant of the left breast (cT2N0M0) was established. Subsequently, a left breast-conserving modified radical mastectomy was performed.

Hematoxylin and eosin as well as immunohistochemical (IHC) stains were conducted on 3-mm formalin-fixed, paraffin-embedded tissue sections. The pathological diagnosis at our facility indicated nodular infiltrative growth of lymphoid tissue in the left breast, with a consideration towards follicular lymphoma/mucosa-associated lymphoid tissue (MALT) lymphoma, while this could not be definitively excluded (Fig. 2I). Therefore, a referral to a specialized healthcare facility was recommended. Notably, examination of the 3 anterior sentinel lymph nodes in the left axilla revealed absence of any tumor tissue.

Further consultation with Shanghai Ruijin Hospital confirmed the pathologic diagnosis of T-lymphoblastoid lymphoma in the left breast. IHC analysis employing a panel of antibodies revealed the following results: TdT(+), Myeloperoxidase (sparse+), CD117 (partially+), CD3(+), CD5(+), CD7(+), CD10(+), CD43(+), C-MYC (~80%+), CD79a (weakly+), BCL-2(+), CD20(−), BCL-6(−), CyclinD1(−), SOX-11(−), IgD(−), MUM-1(−), Kappa(−), Lambda(−), CD56(−), CD4(−), CD2(−), CD8(−), CD34(−), and P53 (~95%+, moderate intensity), along with positivity for CD21 (follicular dendritic cell [FDC+]) and CD23 (FDC+), while programmed death-ligand 1 (combined positive score [CPS] < 1) and Ki67 (~85%+) were also assessed; in situ hybridization for Epstein-Barr virus Encoded RNA yielded a negative result. Evaluation of the left axillary lymph nodes did not reveal any concerning findings.

### 3.3. Synthesis of literature

Fluorescence in situ hybridization analysis utilizing MALT1(18q21) breakage probe, BIRC3/MALT1 fusion probe, and TP53(17p13) deletion probe was negative.

Additionally, a bone marrow biopsy exhibited essentially normal hematopoietic cell proliferation across the 3 lineages with no evident heterogeneous component. A comprehensive genetic screening encompassing 84 genes for peripheral T-lineage lymphoma did not reveal any clinically significant genetic variants.

In the study, a total of 11 cases were included, consisting of 6 cases meeting the criteria with comprehensive clinical data reported in English literature,^[4-9]^ 4 cases reported in Chinese literature,^[10-13]^ and 1 case from our own research (Table 1). Notably, all 11 cases under scrutiny involved female patients, with an average age of 31.81 ± 11.97 years. Among these cases, 5 exhibited bilateral breast involvement, while the remaining 6 manifested unilateral breast affliction, divided equally between the left and right breasts. One case solely implicated the breast, whereas 10 cases demonstrated extra-mammary infiltration (including 3 instances confined to axillary lymph nodes). IHC evaluation revealed positivity for TdT and CD3 in 9 cases presenting with breast lesions, with 5 cases showing evidence of bone marrow infiltration. 6 cases underwent breast ultrasonography, 6 cases underwent mammography, 1 case underwent MRI examination, and 2 cases underwent PET-CT examination. The breast masses had a maximum diameter ranging from approximately 1.7 cm to 4.4 cm. Treatment strategies predominantly centered around diverse forms of chemotherapy (comprising 4 Cyclophosphamide, Doxorubicin, Vincristine, Prednisone regimen, 3 Hype-CVAD protocol, 3 other variants, and 1 unspecified approach), occasionally complemented by combined radiotherapy and chemotherapy. Additionally, certain cases received a combination of radiotherapy and intrathecal drug therapy to avert central nervous system infiltration.

**Table 1:** Reported cases of T-LBL involving breast.

No.	Author	Age	Initial presentation and symptom	Breast	Extramammary involvement	IHC profile	Bone marrow biopsy	Genetic abnormality	Treatment	Follow-up
1	Schwartz ^[4]^	21	Dyspnea and chest pain.	R	Hepatomegaly, mediastinal mass	CD1, CD2, CD3, CD5, CD6, TdT	−	N/A	Chemo (CHOP)	N/A
2	Yumuk ^[5]^	20	Weight loss, night sweats, fatigue, left breast masse	L	Lympadenopathy, bilateral pleural effusion, pericardial effusion and hepatomegaly	CD3	+	N/A	Chemo (6 cycles of CHOP) + CNS prophylaxis + RT (brain)	12M
3	Vakiani ^[6]^	41	Asymptomatic	Bi	Mediastinal mass	TdT, CD99, CD3, CD7, focally CD5	−	T(3,11)(q21; p12-13)	Chemo (GMALL protocol), RT (brain, mediastinum)	24M
4	Gallagher ^[7]^	38	Fatigue, progressive enlargement of bilateral breast masses	Bi	Indurated dermal plaque	CD3, CD5, CD4, CD8, CD10, TdT	−	N/A	Chemo (CHOP) + ASCT + CNS prophylaxis + RT(TBI)	1 Phase CR + 20M
5	Xiao-fan Liu ^[10]^	19	Right breast mass, fever	R	Neck, perihepatic and bilateral axillary adenopathy mediastinal mass, pericardial and pleural effusion	CD3, CD45RO	+	bcr-abl p190-p210-	Chemo (Hype-CVAD) + CNS prophylaxis	5M
6	Liang Li ^[11]^	32	Left breast mass	L	None	CD3, CD5, CD43, TdT, CD79α(80%), LAT, bcl-2, CD10, Ki-67 (40%)	−	N/A	N/A	4M
7	Gang-gang Wang ^[12]^	17	Left breast mass	Bi	Left axillary adenopathy	CD3, TdT, bcl-2, Ki-67 (70%),	+	N/A	Chemo (modified BMF-90)	12M
8	Katagiri ^[8]^	54	Double vision	Bi	A right eyelid tumor	cyCD3, TdT	+	t(7;14)(p15;q32)	Chemo (L‐aspar‐aginase) + CBT	2 Phase CR
9	Iqbal ^[9]^	33	Asymptomatic	R	Mediastinal mass, pericardial and pleural effusions	CD1a, CD2, CD3, CD5, CD7, CD8, CD10(dim), CD45, CD99, BCL2, TdT, c-MYC, Ki-67,	+	9p LOH, 13q deletion, TCR+	Chemo (hyper-CVAD)	N/A
10	Xiao-die Zhou ^[13]^	30	Left breast masse	L	Left axillary adenopathy	CD3, CD5, CD1, CD99, CD34, TDT, Ki-67,	−	TCR+	Tumor resection + Chemo (CHOP)	6M
11	Present case	45	Left breast masse	Bi	Left axillary adenopathy	See result	−	–	See Figure 1	See Figure 1

CD = cluster of differentiation, IHC = immunohistochemistry, TdT = terminal deoxynucleotidyl transferase, T-LBL = T-cell lymphoblastic lymphoma.

## 4. Discussion

Breast lymphoma is a rare hematological neoplasm that arises from the lymphoid tissue within the breast, with an estimated incidence ranging from ~0.04% to 0.7%. Predominantly classified as non-Hodgkin’s lymphoma, the scarcity of breast lymphomas may be attributed to the relatively limited presence of lymphoid tissue in this anatomical site.^[14]^ T-cell lymphoblastoid lymphoma (LBL) represents a subset of non-Hodgkin’s lymphoma characterized by high aggressiveness, primarily affecting adolescents and young males. It constitutes around 3% to 4% of non-Hodgkin’s lymphomas in adults and ~40% of cases in pediatric populations, often exhibiting mediastinal and bone marrow infiltration.^[5,9,15]^ This entity, known for its aggressive behavior, malignancy, and heterogeneity, typically originates from immature precursor T lymphocytes. Notably, T-LBL presenting in the breast is exceedingly rare, further underscoring the unique nature of this pathological entity.^[15]^

Breast T-LBL is typically diagnosed through biopsy or postoperative pathology, characterized by medium-sized lymphoblasts exhibiting round, oval, or convoluted nuclei, finely dispersed nuclear chromatin, inconspicuous or small nucleoli, scanty basophilic cytoplasm, frequent mitoses, and regions of necrosis. Distinguishing features that differentiate it from B-lymphoblastic lymphoma require immunophenotypic analysis.^[15]^ This lymphoma subtype, predominantly of T-lymphoblastic origin comprising about 80% of cases, commonly exhibits positivity for TdT, CD3, CD7 in 90% of instances, with approximately half showing dual positivity for CD4 and CD8, and frequent expression of CD10, CD34, CD1a, and CD99.^[15]^ Notably, CD10, CD34, CD1a, and CD99 markers are commonly detected.

Our investigation challenges prior notions about the sidedness of breast lymphomas by revealing a predominant bilaterality in cases of T-lymphoblastoid lymphomas.^[15-18]^ The bilateral involvement is linked to the notably aggressive nature of this disease. Mediating mediastinal involvement, these lymphomas are often associated with pericardial or pleural effusions, necessitating crucial thoracic imaging for assessing mediastinal mass presence and evaluating the risk of pericardial tamponade. While infiltration of peripheral lymph nodes, particularly cervical nodes, is common, central nervous system involvement is relatively infrequent (<5% of patients).^[19]^ Bone marrow invasion is observed in ~15% to 20% of cases, typically characterized by active or markedly active bone marrow nucleated cell hyperplasia with primitive cell hyperplasia, although this occurrence is less than a quarter of cases. Utilizing multiparametric flow cytometry, T-LBL typically shows expression of T-cell specific markers such as CD3, and often exhibits CD38, CD7, CD99, cTdT, CD2 markers.^[15]^

With the rapid advancement of diverse detection methodologies, the exploration of the molecular underpinnings of T-LBL has progressed from the cellular realm to the genetic domain. Various genetic abnormalities have been distinctly elucidated, encompassing antigen receptor gene rearrangements (e.g., clonal rearrangement of TCRαβ chain genes), chromosomal aberrations (e.g., translocations, deletions, and rearrangements), oncogene inactivation (e.g., PTEN, FBXW7, and BIM), and oncogene activation (e.g., Notch1, C-MYC, NKX3-1, among others), epigenetic modifications (e.g., PHF6), alongside the delineation of activation mechanisms for several signaling pathways such as GR-p38-MAPK/JNK, PI3K-Akt, elucidating further insights.^[20-22]^ In the current investigation, 5 cases exhibited genetic testing abnormalities.^[6,8-10,13]^

Breast imaging for T-LBL of the breast mirrors that of other breast lesions, encompassing techniques such as ultrasound, molybdenum target imaging, and MRI. However, given the rarity of breast lymphoma, particularly T-LBL, there exists a scarcity of literature documenting its imaging characteristics. While the imaging features of breast T-LBL share similarities with breast lymphoma, they are not entirely identical. Mammography and ultrasonography findings in breast lymphoma often lack distinct characteristics. Ultrasonographic patterns in breast lymphoma may range from hypoechoic or isoechoic to markedly hyperechoic, or showcase diffuse infiltrative echogenicity with well-defined or ill-defined margins, accompanied by abundant blood flow signals detected on Doppler imaging.^[18,23,24]^ Within our study cohort, 6 patients underwent ultrasonography, with 5 of them^[4,5,7,9,13]^ exhibiting a breast mass characterized by primarily cystic solidity (i.e., mixed echoes), while one displayed heterogeneous hypoechoic properties with poorly delineated borders. A recent case study demonstrates that its ultrasound presentation consists of heterogeneous hypoechoic areas in the skin layer and subcutaneous tissue of the upper outer quadrants of both breasts, with contrast-enhanced ultrasound revealing rapid homogeneous hyperenhancement of the masses and a small non-enhanced area in the center.^[25]^ Mammographic presentations of breast lymphoma typically reveal solid masses devoid of calcification, although they may also manifest as multiple masses or diffuse asymmetric densification alongside skin thickening. Notably, up to 13% of breast lymphomas may demonstrate no discernible abnormalities on mammograms.^[24,26]^ In our investigation, 6 patients underwent molybdenum imaging screening,^[6,7,9,10,13]^ revealing varied presentations: one showcased a solitary dense mass, another displayed asymmetric densification, 2 had multiple solid masses, and 2 exhibited unremarkable findings, aligning with existing literature on breast lymphoma imaging manifestations.

Several MRI studies have previously explored breast lymphoma, yet literature describing MRI findings specifically for breast T-LBL is limited. Reports on MRI findings of T-lymphoblast lymphoma outside the breast are sparse. Erdem ^[27]^ documented lumbar epidural T-LBL, while Gupta et al^[28,29]^ described pituitary T-LBL with isosignal on T1-weighted imaging, slightly low signal on T2-weighted imaging (attributed to cellular densification), and marked heterogeneous enhancement, findings akin to the current report. In breast lymphoma, MRI typically depicts one or multiple masses in the breast with equal or low signal intensity on T1-weighted imaging, high or slightly high signal intensity on T2-weighted imaging, low ADC, and time-signal intensity curve (TIC) exhibiting plateau or outflow patterns (type II or III) relative to breast parenchymal signal.^[30-32]^ In our study, 1 patient underwent MRI scanning with enhancement, revealing features consistent with breast lymphoma, including restricted diffusion on DWI, markedly low signal on ADC map with ADC values below 0.6 × 10^−3^ mm^2^/s. These MRI findings are likely linked to tumor pathology, characterized by abundant and densely packed intracellular cells with minimal extracellular interstitial space. The heterogeneous enhancement post-enhancement likely reflects the presence of necrotic areas within the tumor. The plateau-type time-signal enhancement curve is a pattern consistent with other lymphoma types in the breast, closely aligning with the tumor’s pathological characteristics. Another patient underwent whole body diffusion-weighted whole body imaging with background signal suppression (DWIBS), a technique akin to a whole-body “PET-like” scan, primarily used for hematologic disease and metastatic tumor screening, offering significant value in medical imaging.^[33]^ DWIBS, an advanced form of traditional DWI incorporating Short Tau Inversion Recovery technology, assesses water molecule diffusion characteristics in various human tissues by quantifying the ADC. DWIBS can evaluate lymph node and extra-nodal tissue involvement in lymphoma patients, with significantly lower ADC values observed in affected patients compared to normal lymph nodes. Moreover, it serves as a valuable tool for assessing lymphoma treatment efficacy, akin to PET-CT but utilizing distinct imaging principles and scanning modes.^[34]^ In one case from our study undergoing MRI DWIBS examination,^[8]^ findings revealed a diffusion-restricted multilocular intramammary mass with markedly low ADC values, indicating involvement of multiple extramammary organs and the lymphatic system. Consequently, lower ADC values may aid in the diagnosis of T-LBL of the breast.

Upon confirming the diagnosis of T-lymphoblastoid lymphoma, a PET-CT scan is imperative to assess extranodal organ involvement. Lymphoma lesions typically exhibit high ^18^F-FDG uptake, with reported median SUVmax values exceeding 10 for T-lymphoblastoid lymphoma. A case highlighted in our literature review demonstrated significant uptake in the bilateral breast, mediastinum, and retroperitoneum, although specific SUVmax values were not provided. In contrast, a case we presented showcased postoperative changes in the left breast with increased uptake in the left axillary lymph node, despite negative pathological findings. Notably, a region of slightly elevated uptake in the right breast corresponded to an area of ADC decompensation observed on MRI. PET-CT scans are pivotal not only for assessing treatment efficacy and patient prognosis but also for guiding therapeutic decisions.^[35-38]^ Studies have shown that PET-CT scans pre- and post-allogeneic hematopoietic stem cell transplantation (HSCT) serve as prognostic indicators for overall survival and disease-free survival in acute leukemia patients. In our case report, post-treatment PET-CT scans did not reveal any areas of high FDG uptake, including the previously noted region in the right breast, suggesting resolution of lymphoma infiltration. However, due to the lack of pretreatment pathological diagnosis for the right breast lesion, the lymphomatous involvement could not be confirmed initially.

Following a T-LBL diagnosis in the breast, mastectomy does not confer survival benefits, as the post-mastectomy survival rate is low.^[39]^ Treatment should be individualized based on the systemic nature of the disease, encompassing induction, consolidation, and maintenance phases.^[15]^ Standard induction regimens such as ALL-like regimens, BFM-90/95, cyclophosphamide, vincristine, doxorubicin, dexamethasone (intensive regimen), and pediatric ALL-like regimens are commonly employed. Intrathecal chemotherapy for central nervous system prophylaxis is essential, utilizing agents like dexamethasone, methotrexate, and cytarabine. Mediastinal radiotherapy serves as an important tool to prevent mediastinal recurrence. Recent research has highlighted HSCT as a viable consolidation treatment option for T-LBL patients in first complete remission without thoracic involvement. Both auto-HSCT and matched sibling HSCT have shown promise in improving survival rates for patients in first complete remission.^[40]^ Among the 11 patients included in this study, 2 cases^[4,9]^ were lacking follow-up documentation. One case^[13]^ underwent tumor resection followed by chemotherapy and passed away after a 6-month follow-up period. In another instance,^[11]^ details of treatment were missing, and the patient expired after a 4-month follow-up. A single case^[12]^ received a modified BMF-90 chemotherapy regimen and exhibited successful recovery after a 12-month follow-up. Additionally, a patient in case^[8]^ underwent combined chemotherapy and umbilical cord blood transplantation, achieving a favorable outcome at the conclusion of follow-up. Furthermore, 5 cases^[5-7,10]^ underwent systemic chemotherapy with central nervous system prophylaxis (comprising 2 cases with HSCT and 2 cases with mediastinal or total body irradiation), all exhibiting positive outcomes at the end of follow-up, thereby showcasing the effectiveness of diverse combined therapeutic approaches.

## 5. Conclusions

Ultrasound and molybdenum target examination of breast T-lymphoblastoid lymphoma lack specificity. In MRI, the identification of single or multiple breast masses exhibiting low ADC values and type II or III TIC enhancement curves in both breasts should prompt recommendation for puncture biopsy to prevent misdiagnosis as breast cancer, avoidance of unnecessary mastectomy, and delay in initiating systemic treatment. Patients with suspected breast T-lymphoblastoid lymphoma should undergo additional bone marrow aspiration biopsy to differentiate it from T-ALL. PET-CT examinations conducted before and after treatment play a pivotal role in the diagnosis and management of T-lymphoblastoma. Imaging plays a crucial role in formulating diagnostic and treatment strategies, while confirmation of diagnosis still requires immunohistochemistry and cytogenetic tests. The management of T-LBL necessitates a comprehensive approach involving systemic chemotherapy, central nervous system prophylaxis, and stem cell transplantation. Future efforts should focus on conducting prospective studies to establish more precise noninvasive diagnostic protocols and to optimize evidence-based comprehensive treatment strategies for involvement of this rare site.

## Acknowledgments

We are grateful to the patient.

## Author contributions

**Conceptualization:** Wei Bian, Yi Yang.

**Data curation:** Wei Bian, Jun Yuan, Yufeng Fei, Xu Zhang, Sili Jin.

**Formal analysis:** Jun Yuan, Jinjuan Feng.

**Methodology:** Jun Yuan.

**Writing – original draft:** Wei Bian.

**Writing – review & editing:** Wei Bian.
